# Supplementary material for: A UMLS-based spell checker for natural language processing in vaccine safety
Source: BMC Med Inform Decis Mak. 2007 Feb 12;7:3. doi: 10.1186/1472-6947-7-3 (PMC1805499; doi:10.1186/1472-6947-7-3)
Supplement: Additional file 13 — RAP application directory. Sets up directory for RAP (RDF application for PHP) source code [file 1472-6947-7-3-S13.gz › rap/doc/phpdoc/__filesource/fsource_default__vocabularyFOAF_C.php.html]

xml version="1.0" encoding="iso-8859-1"?


File Source for FOAF\_C.php


# Source for file FOAF\_C.php

Documentation is available at FOAF\_C.php

```
1. <?PHP
2. // ----------------------------------------------------------------------------------
3. // Friend of a Friend (FOAF) Vocabulary (Resource)
4. // ----------------------------------------------------------------------------------
5. // Version                   : 0.9
6. // Authors                   : Tobias Gau� (tobias.gauss@web.de)
7. //
8. // Description               : Wrapper, defining resources for all terms of the
9. //                               Friend of a Friend project (FOAF).
10. //                               For details about FOAF see: http://xmlns.com/foaf/0.1/.
11. //                                Using the wrapper allows you to define all aspects of
12. //                             the vocabulary in one spot, simplifing implementation and
13. //                             maintainence.
14. //
15. // ----------------------------------------------------------------------------------
16. // History:
17. // 11-08-2003                 : Initial version
18. // ----------------------------------------------------------------------------------


28. class FOAF{


31. function AGENT()
32. {
33. return  new Resource(FOAF_NS . 'Agent');

35. }

37. function DOCUMENT()
38. {
39. return  new Resource(FOAF_NS . 'Document');

41. }

43. function GROUP()
44. {
45. return  new Resource(FOAF_NS . 'Group');

47. }

49. function IMAGE()
50. {
51. return  new Resource(FOAF_NS . 'Image');

53. }

55. function ONLINE_ACCOUNT()
56. {
57. return  new Resource(FOAF_NS . 'OnlineAccount');

59. }

61. function ONLINE_CHAT_ACCOUNT()
62. {
63. return  new Resource(FOAF_NS . 'OnlineChatAccount');

65. }

67. function ONLINE_ECOMMERCE_ACCOUNT()
68. {
69. return  new Resource(FOAF_NS . 'OnlineEcommerceAccount');

71. }

73. function ONLINE_GAMING_ACCOUNT()
74. {
75. return  new Resource(FOAF_NS . 'OnlineGamingAccount');

77. }

79. function ORGANIZATION()
80. {
81. return  new Resource(FOAF_NS . 'Organization');

83. }

85. function PERSON()
86. {
87. return  new Resource(FOAF_NS . 'Person');

89. }

91. function PERSONAL_PROFILE_DOCUMENT()
92. {
93. return  new Resource(FOAF_NS . 'PersonalProfileDocument');

95. }

97. function PROJECT()
98. {
99. return  new Resource(FOAF_NS . 'Project');

101. }

103. function ACCOUNT_NAME()
104. {
105. return  new Resource(FOAF_NS . 'accountName');

107. }

109. function ACCOUNT_SERVICE_HOMEPAGE()
110. {
111. return  new Resource(FOAF_NS . 'accountServiceHomepage');

113. }

115. function AIM_CHAT_ID()
116. {
117. return  new Resource(FOAF_NS . 'aimChatID');

119. }

121. function BASED_NEAR()
122. {
123. return  new Resource(FOAF_NS . 'based_near');

125. }

127. function CURRENT_PROJECT()
128. {
129. return  new Resource(FOAF_NS . 'currentProject');

131. }

133. function DEPICTION()
134. {
135. return  new Resource(FOAF_NS . 'depiction');

137. }

139. function DEPICTS()
140. {
141. return  new Resource(FOAF_NS . 'depicts');

143. }

145. function DNA_CHECKSUM()
146. {
147. return  new Resource(FOAF_NS . 'dnaChecksum');

149. }

151. function FAMILY_NAME()
152. {
153. return  new Resource(FOAF_NS . 'family_name');

155. }

157. function FIRST_NAME()
158. {
159. return  new Resource(FOAF_NS . 'firstName');

161. }

163. function FUNDED_BY()
164. {
165. return  new Resource(FOAF_NS . 'fundedBy');

167. }

169. function GEEKCODE()
170. {
171. return  new Resource(FOAF_NS . 'geekcode');

173. }

175. function GENDER()
176. {
177. return  new Resource(FOAF_NS . 'gender');

179. }

181. function GIVENNAME()
182. {
183. return  new Resource(FOAF_NS . 'givenname');

185. }

187. function HOLDS_ACCOUNT()
188. {
189. return  new Resource(FOAF_NS . 'holdsAccount');

191. }

193. function HOMEPAGE()
194. {
195. return  new Resource(FOAF_NS . 'homepage');

197. }

199. function ICQ_CHAT_ID()
200. {
201. return  new Resource(FOAF_NS . 'icqChatID');

203. }

205. function IMG()
206. {
207. return  new Resource(FOAF_NS . 'img');

209. }

211. function INTEREST()
212. {
213. return  new Resource(FOAF_NS . 'interest');

215. }

217. function JABBER_ID()
218. {
219. return  new Resource(FOAF_NS . 'jabberID');

221. }

223. function KNOWS()
224. {
225. return  new Resource(FOAF_NS . 'knows');

227. }

229. function LOGO()
230. {
231. return  new Resource(FOAF_NS . 'logo');

233. }

235. function MADE()
236. {
237. return  new Resource(FOAF_NS . 'made');

239. }

241. function MAKER()
242. {
243. return  new Resource(FOAF_NS . 'maker');

245. }

247. function MBOX()
248. {
249. return  new Resource(FOAF_NS . 'mbox');

251. }

253. function MBOX_SHA1SUM()
254. {
255. return  new Resource(FOAF_NS . 'mbox_sha1sum');

257. }

259. function MEMBER()
260. {
261. return  new Resource(FOAF_NS . 'member');

263. }

265. function MEMBERSHIP_CLASS()
266. {
267. return new Resource(FOAF_NS . 'membershipClass');

269. }

271. function MSN_CHAT_ID()
272. {
273. return  new Resource(FOAF_NS . 'msnChatID');

275. }

277. function MYERS_BRIGGS()
278. {
279. return  new Resource(FOAF_NS . 'myersBriggs');

281. }

283. function NAME()
284. {
285. return  new Resource(FOAF_NS . 'name');

287. }

289. function NICK()
290. {
291. return  new Resource(FOAF_NS . 'nick');

293. }

295. function PAGE()
296. {
297. return  new Resource(FOAF_NS . 'page');

299. }

301. function PAST_PROJECT()
302. {
303. return  new Resource(FOAF_NS . 'pastProject');

305. }

307. function PHONE()
308. {
309. return  new Resource(FOAF_NS . 'phone');

311. }

313. function PLAN()
314. {
315. return  new Resource(FOAF_NS . 'plan');

317. }

319. function PRIMARY_TOPIC()
320. {
321. return  new Resource(FOAF_NS . 'primaryTopic');

323. }

325. function PUBLICATIONS()
326. {
327. return  new Resource(FOAF_NS . 'publications');

329. }

331. function SCHOOL_HOMEPAGE()
332. {
333. return  new Resource (FOAF_NS . 'schoolHomepage');

335. }

337. function SHA1()
338. {
339. return  new Resource (FOAF_NS . 'sha1');

341. }

343. function SURNAME()
344. {
345. return  new Resource (FOAF_NS . 'surname');

347. }

349. function THEME()
350. {
351. return  new Resource(FOAF_NS . 'theme');

353. }

355. function THUMBNAIL()
356. {
357. return  new Resource(FOAF_NS . 'thumbnail');

359. }

361. function TIPJAR()
362. {
363. return  new Resource(FOAF_NS . 'tipjar');

365. }

367. function TITLE()
368. {
369. return  new Resource(FOAF_NS . 'title');

371. }

373. function TOPIC()
374. {
375. return  new Resource(FOAF_NS . 'topic');

377. }

379. function TOPIC_INTEREST()
380. {
381. return  new Resource(FOAF_NS . 'topic_interest');

383. }

385. function WEBLOG()
386. {
387. return  new Resource(FOAF_NS . 'weblog');

389. }

391. function WORK_INFO_HOMEPAGE()
392. {
393. return  new Resource(FOAF_NS . 'workInfoHomepage');

395. }

397. function WORKPLACE_HOMEPAGE()
398. {
399. return  new Resource(FOAF_NS . 'workplaceHomepage');

401. }

403. function YAHOO_CHAT_ID()
404. {
405. return  new Resource(FOAF_NS . 'yahooChatID');
406. }
407. }


413. ?>
```

Documentation generated on Fri, 17 Dec 2004 16:14:53 +0100 by phpDocumentor 1.3.0RC3
